# IS*26*-Mediated Formation of Transposons Carrying Antibiotic Resistance Genes

**DOI:** 10.1128/mSphere.00038-16

**Published:** 2016-04-06

**Authors:** Christopher J. Harmer, Ruth M. Hall

**Affiliations:** School of Molecular Bioscience, The University of Sydney, Sydney, New South Wales, Australia; Swiss Federal Institute of Technology Lausanne

**Keywords:** IS*26*, translocatable unit, transposition, transposons

## Abstract

The IS*26* transposase, Tnp26, catalyzes IS*26* movement to a new site and deletion or inversion of adjacent DNA via a replicative route. The intramolecular deletion reaction produces a circular molecule consisting of a DNA segment and a single IS*26*, which we call a translocatable unit or TU. Recently, Tnp26 was shown to catalyze an additional intermolecular, conservative reaction between two preexisting copies of IS*26* in different plasmids. Here, we have investigated the relative contributions of homologous recombination and Tnp26-catalyzed reactions to the generation of a transposon from a TU. Circular TUs containing the *aphA1a* kanamycin and neomycin resistance gene or the *tet*(D) tetracycline resistance determinant were generated *in vitro* and transformed into *Escherichia coli* recA cells carrying R388::IS*26*. The TU incorporated next to the IS*26* in R388::IS*26* forms a transposon with the insertion sequence (IS) in direct orientation. Introduction of a second TU produced regions containing both the *aphA1a* gene and the *tet*(D) determinant in either order but with only three copies of IS*26*. The integration reaction, which required a preexisting IS*26*, was precise and conservative and was 50-fold more efficient when both IS*26* copies could produce an active Tnp26. When both ISs were inactivated by a frameshift in *tnp26*, TU incorporation was not detected in *E. coli* recA cells, but it did occur in *E. coli recA*^+^ cells. However, the Tnp-catalyzed reaction was 100-fold more efficient than RecA-dependent homologous recombination. The ability of Tnp26 to function in either a replicative or conservative mode is likely to explain the prominence of IS*26*-bounded transposons in the resistance regions found in Gram-negative bacteria.

**IMPORTANCE** In Gram-negative bacteria, IS*26* recruits antibiotic resistance genes into the mobile gene pool by forming transposons carrying many different resistance genes. In addition to replicative transposition, IS*26* was recently shown to use a novel conservative movement mechanism in which an incoming IS*26* targets a preexisting one. Here, we have demonstrated how IS*26*-bounded class I transposons can be produced from translocatable units (TUs) containing only an IS*26* and a resistance gene via the conservative reaction. TUs were incorporated next to an existing IS*26*, creating a class I transposon, and if the targeted IS*26* is in a transposon, the product resembles two transposons sharing a central IS*26*, a configuration observed in some resistance regions and when a transposon is tandemly duplicated. Though homologous recombination could also incorporate a TU, Tnp26 is far more efficient. This provides insight into how IS*26* builds transposons and brings additional transposons into resistance regions.

## Abstract (precis)

In Gram-negative bacteria, IS*26* recruits antibiotic resistance genes into the mobile gene pool by forming transposons carrying many different resistance genes. In addition to replicative transposition, IS*26* was recently shown to use a novel conservative movement mechanism in which an incoming IS*26* targets a preexisting one. Here, we have demonstrated how IS*26*-bounded class I transposons can be produced from translocatable units (TUs) containing only an IS*26* and a resistance gene via the conservative reaction. TUs were incorporated next to an existing IS*26*, creating a class I transposon, and if the targeted IS*26* is in a transposon, the product resembles two transposons sharing a central IS*26*, a configuration observed in some resistance regions and when a transposon is tandemly duplicated. Though homologous recombination could also incorporate a TU, Tnp26 is far more efficient. This provides insight into how IS*26* builds transposons and brings additional transposons into resistance regions.

## INTRODUCTION

**FIG 1:**
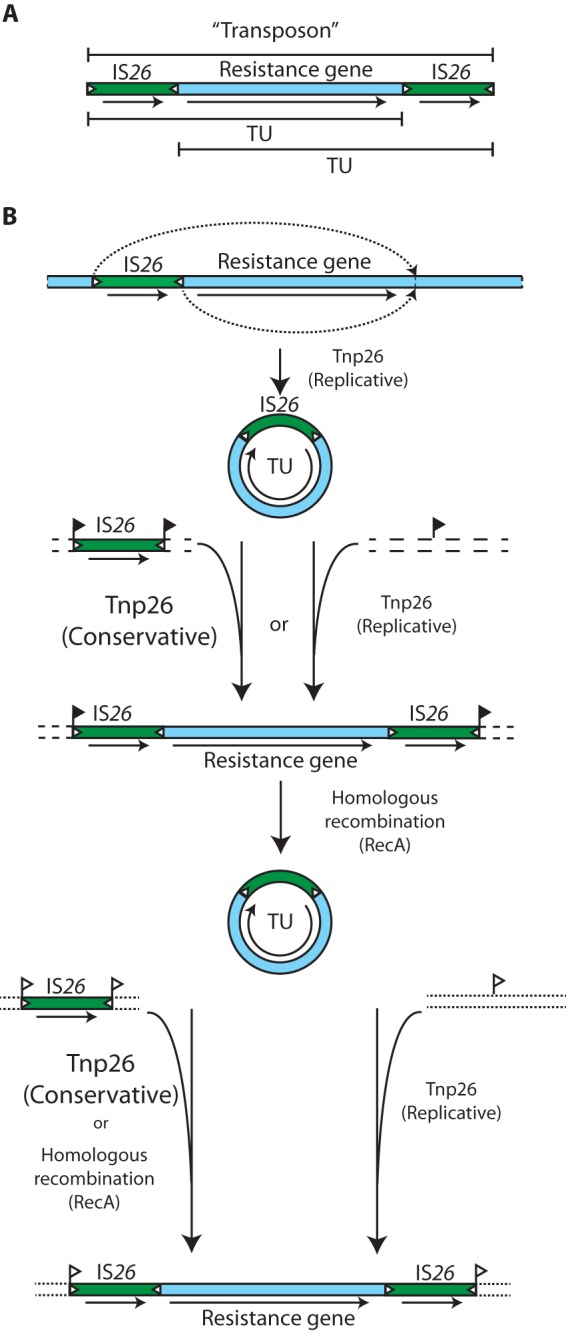
Building IS*26*-bounded transposons. (A) Schematic of a typical IS*26*-bounded class I transposon. IS*26* (green box) and the 14-bp inverted repeats of IS*26* (open triangles) are indicated. An arrow indicates the position and orientation of *tnp26*. The extent of the compound transposon and two alternate translocatable units (TUs) are shown above and below the schematic representation. (B) Two pathways to IS*26*-mediated formation of compound transposons. The relative frequency of the Tnp26-mediated and RecA-dependent reactions is indicated by the size of the label. Eight-base-pair direct repeats of the target sequence are indicated by a flag.

Most class I transposons in Gram-negative bacteria are bounded by two copies of IS*26*. In recent years, the precise origins of several antibiotic resistance genes have been uncovered. In two cases, the *bla*_SHV_ and *oqxAB* genes have been picked up from the chromosome of a *Klebsiella pneumoniae* strain by IS*26* and formed into transposons, which became part of the mobile gene pool (1, 2) (Fig. 1A). IS*26* is known to move via a replicative mechanism and can use this mechanism (3, 4) to cause the deletion of sequence immediately adjacent to one of its ends (Fig. 1B) or invert adjacent DNA. The deletion reaction would create a circular product that includes a single copy of IS*26* together with the deleted DNA segment. As this product is unable to replicate, it would be lost unless it was reincorporated. However, we have recently invoked this form, which we named a translocatable unit (TU), as the unit of movement for IS*26*-flanked transposons (5). To create a transposon, the TU would either be incorporated at a new location via replicative transposition or incorporated next to a preexisting IS*26* using homologous recombination or a Tnp26-catalyzed conservative reaction as shown in Fig. 1B. We recently demonstrated that the IS*26*-encoded transposase, Tnp26, can catalyze a reaction involving two copies of IS*26* that is conservative, i.e., neither the IS*26* nor the target site is duplicated (5). It has been shown that replicative transposition occurs at least 50-fold less frequently than the IS*26*-targeted conservative reaction (5). However, the efficiency of the conservative reaction relative to RecA-dependent homologous recombination is not known.

**FIG 2:**
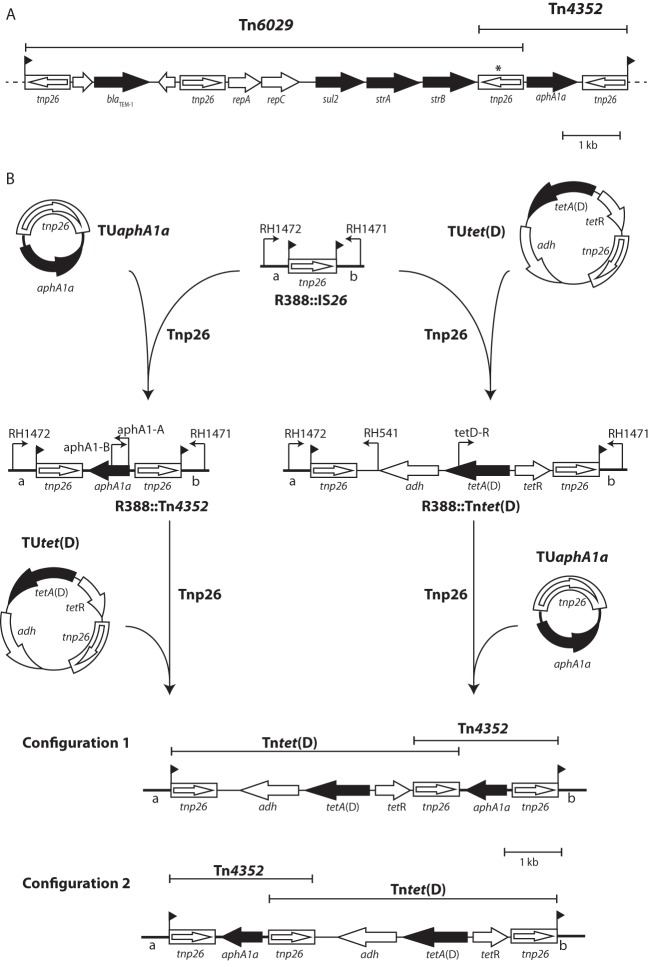
Formation of complex antibiotic resistance regions. (A) Structure of Tn*6026*. (B) Building transposons using TUs derived from Tn*4352* and Tn*tet*(D). The origin of each IS*26*-bounded structure is shown above. Genes and open reading frames are shown as arrows indicating the direction of transcription. Genes conferring antibiotic resistance are black. IS*26* elements are shown as open boxes with an arrow indicating the position and orientation of *tnp26*.

Once a transposon has been generated, it can then move to a new location via a TU intermediate (Fig. 1B, bottom). Though in rare cases Tnp26 can excise a TU, in other cases this does not occur (6), and generation of a TU from a preexisting transposon would necessarily occur via homologous recombination. Again, the TU can be incorporated next to an existing IS*26* using either homologous recombination or the conservative Tnp26-catalyzed mechanism. Repetition of the incorporation process with a second TU should lead to the formation of overlapping transposons such as Tn*4352* and Tn*6029* as shown in Fig. 2A. This structure, found in an IncHI2 plasmid from *Salmonella enterica*, is flanked by a target site duplication of 8 bp (Fig. 2A) (7).

In this study, we investigated the role of the conservative Tnp26 reaction in the generation of transposons from TUs. TUs were generated *in vitro* containing the *aphA1a* kanamycin and neomycin resistance determinant or the *tet*(D) tetracycline resistance determinant and used to generate transposons, which were then built into a multiple resistance region containing overlapping IS*26*-bounded transposons. The efficiency of the Tnp26-catalyzed reaction was compared to that of homologous recombination.

## RESULTS

### IS*26*-mediated incorporation of a TU.

To demonstrate targeted incorporation of a TU adjacent to an IS*26*, the circular form of TU*aphA1a* containing only an IS*26* and a 1,040-bp fragment that includes *aphA1a* (Km^r^) was generated *in vitro* from Tn*4352* as described in Materials and Methods. To ensure that any reaction was catalyzed by Tnp26, and not the result of RecA-dependent homologous recombination, the TU was introduced into the RecA-deficient *Escherichia coli* strain UB1637 (Sm^r^) containing R388::IS*26* (contains the *dfrB2* gene conferring resistance to trimethoprim [Tp^r^]). Transformation of cells with 1 µg of the circular TU yielded an average of 156 Km^r^ Sm^r^ Tp^r^ transformants per µg of TU in four independent experiments (Table 1). UB1637 cells containing R388 devoid of IS*26* were also transformed with 1 µg of TU*aphA1a* to determine the frequency of TU incorporation when IS*26* was not present in the recipient. No kanamycin-resistant transformants were recovered from three independent experiments (Table 1), indicating that IS*26* is required as a target.

**TABLE 1:** Frequency of TU incorporation in a *recA* background

TU	Plasmid in UB1637	No. of transformants/µg of TU^a^	
		Values for individual experiments^b^	Avg value
TU*aphA1a*	R388::IS*26*	166, 153, 142, 162	156
	R388	0, 0, 0	0
TU*tet*(D)	R388::IS*26*	7, 9, 5	7
	R388	0, 0, 0	0
	R388::Tn*4352*	5, 11, 12	9
TU*aphA1a*	R388::Tn*tet*(D)	148, 176, 161	162

a Km^r^ Tp^r^ transformants for TU*4352* or TU*4352*B transformed into cells containing R388::IS*26*, Tc^r^ Tp^r^ transformants for TU*tet*(D) transformed into cells containing R388::IS*26*, Km^r^ Tc^r^ Tp^r^ for TU*4352* or TU*4352*B transformed into cells containing R388:TU*tet*(D) and for TU*tet*(D) transformed into cells containing R388::Tn*4352*.

b Four experiments were performed for TU*aphA1a* and UB1637-R388::IS*26*, and three experiments were performed for all other combinations of TU and plasmid.

Plasmid DNA from 15 transformants (5 from each of 3 replicates) was amplified with the R388 backbone primers RH1471 and RH1472 combined with outward-facing primers aphA1-A and aphA1-B in the *aphA1a* fragment (Fig. 2B). This screening confirmed that, in all cases, R388::Tn*4352* had been reformed by incorporation of the TU adjacent to the existing IS*26* in R388::IS*26* (Fig. 2B, top). Sequencing of the PCR amplicons confirmed that the IS*26* elements were always in direct orientation and that this was a precise conservative reaction such that the 8-bp direct repeats flanking the IS*26* in R388::IS*26* now flanked Tn*4352*. Primers RH1471 and RH1472 yielded an amplicon of 3.4 kb in each transformant tested, demonstrating that only a single copy of the TU had inserted adjacent to the IS*26*.

A second 4.3-kb circular TU derived from Tn*tet*(D) (8) (Tc^r^) was incorporated into R388::IS*26*. Transformation with 1 µg of the TU*tet*(D) yielded between five and nine Tc^r^ Tp^r^ transformants in three independent experiments (Table 1). This frequency is lower than that observed for the incorporation of TU*aphA1a*, and this may reflect a lower transformation efficiency due to the larger size of TU*tet*(D). Again, when R388 replaced R388::IS*26*, no tetracycline-resistant transformants were detected in three independent experiments (Table 1). PCR screening (primers RH541 with RH1472 and primers tetD-R with RH1471 [Fig. 2B]) and sequencing confirmed that, in all cases, R388::Tn*tet*(D) had been reformed by incorporation of the TU adjacent to the existing IS*26* via a precise conservative reaction.

### IS*26*-mediated accumulation of resistance genes.

To demonstrate IS*26*-mediated accumulation of resistance genes, TU*tet*(D) was incorporated into R388::Tn*4352*. Transformation of UB1637 cells containing R388::Tn*4352* with 1 µg of the TU*tet*(D) yielded between 5 and 12 Km^r^ Tc^r^ Tp^r^ transformants in three independent experiments (Table 1). Plasmid DNA from five transformants from each of the independent experiments was amplified with the R388 backbone primers RH1471 and RH1472 in combination with outward-facing primers located within Tn*4352* (aphA1-A and aphA1-B) and Tn*tet*(D) (RH541 and tetD-R) to determine the location of the incorporated TU. In nine cases, the *tet*(D) TU had inserted adjacent to the left-hand IS*26* of Tn*4352* (Fig. 2B, configuration 1), and in six cases, it had inserted adjacent to the right-hand IS*26* (Fig. 2B, configuration 2). Sequencing of the PCR amplicons confirmed that the IS*26* elements were all in direct orientation and the 8-bp direct repeats flanking the IS*26* in R388::Tn*4352* now flanked the whole Tn*4352*-Tn*tet*(D) structure.

The reciprocal experiment, in which TU*aphA1a* was incorporated into R388::Tn*tet*(D), was also performed. An average of 162 Km^r^ Tc^r^ Tp^r^ transformants/µg of TU was recovered from three independent experiments. PCR screening of 15 Km^r^ Tc^r^ Tp^r^ transformants (five from each of three independent experiments) again revealed transformants with configuration 1 (eight) and configuration 2 (seven) as shown in Fig. 2B. Again, the number of transformants was approximately 20-fold higher with TU*aphA1a* than with TU*tet*(D).

### Tnp26 is required for targeted TU incorporation.

The requirement for Tnp26 was confirmed by utilizing a TU and target plasmid that cannot produce active Tnp26. *E. coli* UB1637 (*recA*) cells containing R388::IS*26* were first transformed with 1 µg of TU*aphA1a* from pRMH761 to generate a baseline frequency for the incorporation of the Tn*4352*B-derived TU. This yielded an average of 175 Km^r^ Sm^r^ Tp^r^ transformants per µg of TU in three independent experiments (Table 2).

**TABLE 2:** Transformation of *recA*^+^ and *recA* mutant strains carrying R388::IS*26*

TU (transposase)	Plasmid in UB1637	Expt no. or parameter	No. of Km^r^ Tp^r^ transformants/µg of TU for strain:	
			UB1637 (*recA*)	E294 (*recA*^+^)
TU*aphA1a* (Tnp26)	R388::IS*26*	1	100	195
		2	272	126
		3	153	181
		Avg	175	167.3
TU*aphA1a* (Tnp26-FS-L^a^)	R388::IS*26*	1	1	2
		2	2	0
		3	0	0
		4	1	2
		Avg	1	1
TU*aphA1a* (Tnp26)	R388::IS*26*-FS-R^b^	1	2	1
		2	2	2
		3	1	0
		4	1	2
		Avg	1.5	1.3
TU*aphA1a* (Tnp26-FS-L)	R388::IS*26*-FS-R	1	0	1
		2	0	2
		3	0	1
		4	0	2
		5	0	0
		Avg	0	1.5

a IS*26*-FS-L, frameshift mutation in *tnp26* in the TU, producing a truncated 30-aa protein.

b IS*26*-FS-R, frameshift mutation in R388::IS*26 tnp26*.

UB1637 (*recA*) cells containing R388::IS*26*-FS-R with a frameshift truncating Tnp26 were transformed with 1 µg of TU*aphA1a* derived from pRMH990 (6), which contains a variant of Tn*4352*B with a frameshift in *tnp26* that introduces a premature stop codon allowing only 30 amino acids (aa) to be translated. TU incorporation was below the limit of detection, and no Km^r^ Tp^r^ transformants were recovered from five independent transformations (Table 2). Hence, a complete Tnp26 is required for TU incorporation to occur.

To determine whether targeted TU incorporation is possible with only one active Tnp26, the UB1637 strain containing R388::IS*26* was transformed with 1 µg of TU*aphA1a* that cannot produce Tnp26 due to FS-L (FS stands for frameshift mutation). This yielded a total of only four Km^r^ Tp^r^ transformants from four independent experiments (Table 2). In the reciprocal experiment, recipient cells carrying R388::IS*26*-FS-R, which cannot produce Tnp26, were transformed with TU*aphA1a* from pRMH761, which can produce full-length Tnp26. Only a total of six Km^r^ Tp^r^ transformants were recovered from four independent experiments (Table 2). These frequencies are >100-fold lower than when both molecules participating in the reaction can produce Tnp26. Screening of plasmid DNA from the 10 Km^r^ Tp^r^ transformants recovered in these experiments showed that the TU had incorporated adjacent to the existing IS*26* in all instances. This shows that while targeted TU incorporation is possible with only one active Tnp26, the frequency of incorporation is dramatically decreased compared to when both participating molecules can produce Tnp26.

### Is Tnp26-mediated TU incorporation more efficient than RecA-dependent recombination?

TU incorporation via homologous recombination between two copies of IS*26* should be possible. To assess the contribution of RecA-dependent recombination to TU integration, TU incorporation was explored using recombination-proficient *E. coli* E294 cells carrying R388::IS*26*. When both insertion sequences (ISs) produced an active Tnp26, an average of 167 Km^r^ Tp^r^ transformants per µg of TU*aphA1a* were recovered (Table 2), similar to the frequency observed in *recA* cells. The frequency of TU incorporation was >100-fold lower (an average of 1.5 Km^r^ Tp^r^ transformants per µg of TU) when both the TU and the target plasmid contained a frameshift in the *tnp26* gene, i.e., when the reaction relied upon RecA-dependent homologous recombination (Table 2). Hence, while RecA-mediated homologous recombination can, as expected, lead to TU incorporation, an active Tnp26 in each participating molecule is the major contributor to targeted TU incorporation.

## DISCUSSION

The formation of transposons containing resistance genes is a major force in the development of multiply and extensively resistant strains of Gram-negative bacteria. This study has demonstrated that a TU containing only an IS*26* and a resistance gene preferentially inserts adjacent to an existing IS*26* in the same cell, creating an IS*26*-bounded transposon. TUs generated from Tn*4352*, Tn*4352*B, and Tn*tet*(D) were able to incorporate adjacent to an IS*26* in R388. Incorporation of a second TU generated a resistance array with overlapping IS*26*-bounded compound transposons, and the 8-bp direct repeat originally flanking the IS*26* in R388 now flanked the whole structure.

In complex resistance regions, the IS*26*-bounded compound transposons Tn*4352* and Tn*6029* are often seen together (7). We found that in the nine sequenced examples in GenBank, Tn*4352* is always incorporated adjacent to the *strB* end of Tn*6029* (Fig. 2A), suggesting that there may be a preference for a TU to target a particular end of an existing IS*26*-bounded structure. However, we detected no such preference in this study using Tn*4352* and Tn*tet*(D). The TUs generated from Tn*4352* and Tn*tet*(D) both incorporated equally well on the left and right ends of existing IS*26*-bounded structures. Hence, it is possible that the Tn*4352*-Tn*6029* structure observed today originated once and has then disseminated as a single unit into multiple strains and species. Regions containing IS*26*-bounded transposons are increasingly being reported in other chromosomes and plasmids, though homologous recombination, rather than the Tnp26-mediated conservative reaction, is often invoked as the mechanism responsible (9). The role of TUs in disseminating genetic material is becoming more evident, with recent studies reporting the detection of circular TUs containing multiple resistance genes (9, 10).

As in our previous study using two plasmids each carrying an IS*26* (5), Tnp26 was essential for TU incorporation at the site of a preexisting IS*26* in a recombination-deficient background. Targeted TU incorporation could also occur via RecA-dependent homologous recombination in RecA^+^ wild-type cells. However, this reaction makes only a minor contribution, as it occurs at a frequency at least 2 orders of magnitude lower than the same reaction catalyzed by Tnp26. Hence, the ability of IS*26* to participate in both replicative transposition and self-targeted transposition can explain the abundance of IS*26*-bounded transposons in the mobile gene pool. Moreover, the findings of this study should be applicable to other members of the IS*6* family, including IS*257* and IS*1216*, that play a major role in the mobilization of antibiotic resistance genes in staphylococci and enterococci, respectively.

## MATERIALS AND METHODS

### Plasmids and plasmid construction.

The plasmids used in this study are listed in Table 3. pRMH761, pRMH976, pRMH990, R388::IS*26*, R388::IS*26*-FS-R, R388::Tn*4352*, and R388::Tn*4352*B have been described previously (5, 6, 11). pUC19::Tn*tet*(D) was constructed by cloning a 6,038-bp Tn*tet*(D)-containing SacI fragment (bases 134454 to 140495 in GenBank accession number KP276584) from the A/C_2_ plasmid p39R861-4 (8) into SacI-digested pUC19. Tc^r^ transformants were screened via PCR and restriction digestion with PstI and NdeI to confirm the identity and orientation of the insert.

**TABLE 3:** Plasmids used in this study

Plasmid	Description	Resistance phenotype^a^	Reference
pRMH761	8.8-kb BamHI fragment of pRMH760 containing Tn*4352*B cloned into pUC19	Ap Km Nm	5
pRMH976	pRMH761 derivative containing Tn*4352* in *tniA*^b^	Ap Km Nm	6
pRMH990	pRMH761 derivative with frameshifts in both *tnp26*^c^	Ap Km Nm	6
R388::IS*26*	R388 with IS*26*^d^	Su Tp	5
R388::IS*26*-FS-R	R388::IS*26* frameshift mutant^e^	Su Tp	5
R388::Tn*4352*B	R388 containing Tn*4352*B^f^	Km Nm Su Tp	5
R388::Tn*4352*	R388 containing Tn*4352*^f^	Km Nm Su Tp	6
p39R861-4	Type 2 A/C_2_ plasmid carrying Tn*tet*(D)	Cm Fl Su Tc	8
pUC19::Tn*tet*(D)	6.0-kb SacI fragment of p39R861-4 containing Tn*tet*(D) cloned into pUC19^g^	Ap Tc	This study

a Ap, ampicillin; Cm, chloramphenicol; Fl, florfenicol; Km, kanamycin; Nm, neomycin; Su, sulfamethoxazole; Tc, tetracycline; Tp, trimethoprim.

b 1.8-kb central SwaI Tn*4352*B fragment replaced with Tn*4352* from pDGO100.

c Frameshift generated by end filling the BsiWI site and duplicating 116 to 119 bp from the left end of IS*26* as shown in Fig. 5 of Harmer et al. (5).

d IS*26* 8-bp duplication of bases 26745 to 26752 in R388 (GenBank accession no. BR000038).

e Lacks 13 bp (bases 624 to 636 from the left end of IS*26*).

f Tn*4352*B together with 8-bp duplication of bases 26745 to 26752 in R388 (GenBank accession number BR000038).

g Bases 134454 to 140495 from p39R861-4 cloned into pUC19.

### DNA manipulation.

Plasmid DNA was isolated by alkaline lysis, digested, and gel purified as previously described (5). PCR and routine sequencing of PCR products were performed as previously described using published primers RH1471, RH1472, aphA1-A, aphA1-B, RH541, and tetD-R (5, 8, 12). The positions and orientations of the primers are marked in Fig. 2B.

### *In vitro* construction and transformation of a TU.

*Escherichia coli* UB1637 (*recA* Sm^r^) and *E. coli* E294 (Rif^r^) (13) were used as recipients in transformation experiments. The presence of a unique SwaI restriction site in IS*26* was exploited to generate a circular TU *in vitro*. A similar strategy has been used successfully to study IntI-dependent insertion of gene cassettes into integrons (14). A Tn*4352*B- or Tn*4352-*derived TU*aphA1a* was generated *in vitro* by SwaI digestion of pRMH761 (Tn*4352*B) or pRMH976 (Tn*4352*) plasmid DNA extracted from cells grown overnight with kanamycin selection. The 1.8-kb fragment containing *aphA1a* and two partial copies of IS*26* was isolated via gel extraction. One microgram of the 1.8-kb fragment was religated using 40 U of T4 DNA ligase (New England Biolabs) to form a circular TU containing *aphA1a* and a complete copy of IS*26*. Similarly, a 4.3-kb fragment was recovered from pUC19::Tn*tet*(D) plasmid DNA digested with SwaI and religated to form TU*tet*(D). pRMH990, a derivative of pRMH761 with a frameshift in both copies of IS*26*, was similarly used to generate TU*aphA1a* encoding an inactive truncated Tnp26.

One microgram of ligation mixture containing the appropriate TU was electroporated into *E. coli* UB1637 (RecA-deficient) or E294 (RecA-proficient) recipient cells that had been rendered electrocompetent as described previously (15). Electroporation was performed using a BioRad MicroPulser electroporator according to the manufacturer’s instructions. After recovery in 1-ml Luria broth for 90 min at 37°C, transformed cells were plated onto Mueller-Hinton agar supplemented with trimethoprim (10 µg ml^−1^) and either kanamycin (50 µg ml^−1^) to select for transformants containing TU*aphA1a* and/or tetracycline (10 µg ml^−1^) to select for TU*tet*(D).
